# CisMiner: Genome-Wide *In-Silico* Cis-Regulatory Module Prediction by Fuzzy Itemset Mining

**DOI:** 10.1371/journal.pone.0108065

**Published:** 2014-09-30

**Authors:** Carmen Navarro, Francisco J. Lopez, Carlos Cano, Fernando Garcia-Alcalde, Armando Blanco

**Affiliations:** 1 Department of Computer Science and AI, University of Granada, Granada, Spain; 2 Andalusian Human Genome Sequencing Centre (CASEGH), Medical Genome Project (MGP), Sevilla, Spain; 3 Max Planck Institute for Infection Biology, Berlin, Germany; National Institutes of Health, United States of America

## Abstract

Eukaryotic gene control regions are known to be spread throughout non-coding DNA sequences which may appear distant from the gene promoter. Transcription factors are proteins that coordinately bind to these regions at transcription factor binding sites to regulate gene expression. Several tools allow to detect significant co-occurrences of closely located binding sites (cis-regulatory modules, CRMs). However, these tools present at least one of the following limitations: 1) scope limited to promoter or conserved regions of the genome; 2) do not allow to identify combinations involving more than two motifs; 3) require prior information about target motifs. In this work we present CisMiner, a novel methodology to detect putative CRMs by means of a fuzzy itemset mining approach able to operate at genome-wide scale. CisMiner allows to perform a blind search of CRMs without any prior information about target CRMs nor limitation in the number of motifs. CisMiner tackles the combinatorial complexity of genome-wide cis-regulatory module extraction using a natural representation of motif combinations as itemsets and applying the Top-Down Fuzzy Frequent- Pattern Tree algorithm to identify significant itemsets. Fuzzy technology allows CisMiner to better handle the imprecision and noise inherent to regulatory processes. Results obtained for a set of well-known binding sites in the *S. cerevisiae* genome show that our method yields highly reliable predictions. Furthermore, CisMiner was also applied to putative in-silico predicted transcription factor binding sites to identify significant combinations in *S. cerevisiae* and *D. melanogaster*, proving that our approach can be further applied genome-wide to more complex genomes. CisMiner is freely accesible at: http://genome2.ugr.es/cisminer. CisMiner can be queried for the results presented in this work and can also perform a customized cis-regulatory module prediction on a query set of transcription factor binding sites provided by the user.

## Introduction

An organism's DNA encodes the information required for each cell to function. However, a complete description of the DNA sequence of an organism is not enough to reconstruct it. Not only genes (i.e. coding DNA) hold relevant information, but also the rest of non-coding DNA, which orchestrates how each element is related to the rest: under which conditions each gene product is made, and which role it plays in the complex machinery of the cell. There are many steps in the pathway leading from DNA to protein. The initiation of RNA transcription is a very important step in such pathway [1].

Eukaryotic gene control regions consist of a promoter region plus a set of regulatory DNA sequences. Transcription factors (TFs) are regulatory proteins that bind at these regions to specific sequences called transcription factor binding sites (TFBSs) forming complexes that are essential to the initiation of gene transcription. In addition, this TF-DNA interaction is usually coordinated forming *cis*-regulatory modules (CRMs) [2].

Many different approaches address *in silico* CRM detection. However, CRM detection has strong performance limitations due to its combinatorial complexity [3]. Overall, three conceptually different classes of methods can be roughly identified according to an increasing genomic scope [4]: 1) CRM scanners, 2) CRM builders and 3) CRM genome screeners.

**CRM scanners** search for sequences that satisfy a strictly defined CRM model. Their goal is usually to further study well-characterized problems, and the user is required to provide a detailed specification of the studied CRM. Therefore, their application is limited to well-known problems and many parameters are usually required, such as the expected distance between TFBSs, a background model for the sequences, number of target TFBSs, a reduced set of target TFBSs or a window size. Cister [5], Cluster-Buster [6] or Stubb [7] belong to this category. Due to their specificity, their execution times tend to be shorter than other methods with a broader scope.

**CRM builders** extend the scope of the CRM prediction by reducing the number of constraints for the target CRM model. They try to assemble CRMs looking for similar features in a reduced set of related sequences. These methods reduce the sequence search space by two main approaches: 1) limiting the study to gene promoter regions of co-expressed or related genes of interest [8], [ 9]; or 2) focusing on evolutionary conserved regions [10]–[12]. The first type of approaches could miss regulatory elements in less obvious locations, such as those located in introns and far upstream or downstream of genes. Indeed, genome-wide chromatin immunoprecipitation experiments [13] have reported that a significant proportion of TFBSs do not lie in regions immediately upstream of known protein-coding genes [14]–[17]. In addition, comparative genomics revealed that many regulatory elements involved in early vertebrate development lie far from the gene they are thought to regulate [10], [ 18]–[20]. The second type of approaches are based on the evolutionary conservation of regulatory sequences. However, the regulatory sequences have to be similar enough to be aligned, which is often not the case when compared sequences are not closely related. Moreover, experiments on mammalian and *Drosophila* species have shown that between one and two-thirds of identified regulatory sequences are not conserved even between closely related species [10], [ 21]–[23]. Approaches like INSECT [24] and CORECLUST [25] optionally combine these two techniques to reduce the search space.

According to the description in the literature [4], **CRM genome screeners** search through complete genomes for CRMs. In contrast to the previous approaches, genome screeners do not make any assumptions regarding the target CRMs. Therefore, they present a broader applicability but tackle a more complex problem. However, although some methods are considered CRM genome screeners in [4], a genome-wide screening tool that encompasses the whole non-coding DNA of an organism without any restriction has not yet been made available. Some approaches, such as D-light [26], do not restrict the number of genes but limit the screening to the promoter regions of these genes, not considering the rest of the non-coding genome. Approaches like COPS [27] search for co-ocurring TFs in sequences known to be bound *in vivo*, therefore requiring previous knowledge on target TFs and experimental evidence of their binding sites. Other approaches like TraFaC [28], PreMod [11] or EEL [12], also reduce the search space to a set of orthologous sequences or co-regulated genes, and therefore could be considered CRM builders. CisMiner, the methodology we present in this work, can be framed into the CRM genome screeners category.

Independently of their scope, all the aforementioned approaches suffer the computational complexity of CRM detection, which is not only increased by the total length of the sequences to screen, but also by the number of putative TFBSs detected and the size of the target modules. In this sense, some approaches reduce the search space by limiting the number of cooperating transcription factors, usually looking for pairs of co-ocurring transcription factors [29], [ 30]. Although these are easier to identify and have been shown to have biological significance, CRMs can encompass larger sets of transcription factors which relate in a more sophisticated manner [2]. These complex CRMs are overlooked with such approaches.

Perhaps because of its combinatorial complexity, specially when applied genome-wide, the vast majority of available approaches for computational CRM discovery restrict the search space (to a set of co-regulated genes and/or orthologous sequences) or the dimensionality of the problem (number or positioning of TFBSs), overlooking larger CRMs or those located in other non-coding regions of the genome. With the currently available approaches, any researcher aiming to obtain a set of putative cis-regulatory modules in a query organism with no prior information about which transcription factors are involved in the process will need a specific set of motifs as an input.

This might not be likely if the researcher does not have any prior knowledge. Thus, the probability of selecting the adequate subset of TFBSs would be equivalent to the probability of picking a random subset of the given set of TFBSs in an organism, which is very small. The same applies for approaches that use orthologous sequences to a certain gene or a set of co-regulated genes. These approaches are useful for the study of already known processes. However, it is very unlikely that a researcher trying to explore a certain organism without prior knowledge will be able to provide a set of specific motifs and sequences useful for extracting knowledge in a given organism.

On the other hand, CRM prediction tools are usually coupled with TFBS detection tools. Therefore, CRM prediction usually requires a set of position weight matrices (PWMs) as input to first apply a TFBS detection tool to screen the query sequences for a set of putative TFBSs. In this case, the overall performance of CRM genome screeners depends heavily on the quality of the predictions made by the TFBSs detection tool. CisMiner, the tool we propose, is able to perform CRM prediction by either using a computational TFBS prediction tool for whole genome sreening, or a set of already predicted or known TFBSs provided by the user.

Furthermore, information about the exact location and length of regulatory regions is imprecise and uncertain. It is known that CRMs can span hundreds of base pairs, although their exact length has not been assessed. Not only the location and length of CRMs, but also the information about the positions where transcription factors bind to the genome, are still vague and inaccurate. *In-silico* tools for TFBS prediction sometimes surpass the reasonable amount of false positives due to the probabilistic and biological complexity of this problem. The sought sequences are indeed very short (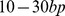) compared to the size of the scanned genomes, and the appearance of sequences which are truly similar to those of real TFBSs but do not represent a regulatory function must be expected as well. This lack of transcription factor sequence specificity may suggest that more complex rules and mechanisms govern the regulation process influenced by transcription factor binding activity [2].

Any computational CRM prediction approach must therefore take these problems into consideration and handle the imprecision and uncertainty unavoidably present in the data. However, although fuzzy techniques are known to outperform classical crisp techniques when dealing with imprecise and noisy data, these approaches are barely used in this field.

In this work we present CisMiner, a fuzzy-based genome-wide CRM screener which overcomes some of the mentioned limitations by allowing to scan whole non-coding genomes for significant combinations of any number of TFBSs. Given a set of TFBSs, CisMiner implements a fuzzy clustering of closely-located TFBSs and analyze these fuzzy sets to obtain combinations of TFBSs which co-occur significantly using the Top-Down Fuzzy Frequent-Pattern Tree algorithm.

To the extent of our knowledge, there are not any available approaches that encompass the whole non-coding genome of an organism and allow a search for highly dimensional CRMs. Therefore, the area of application of CisMiner differs greatly to those of the rest of mentioned approaches. CisMiner is based on performing a fuzzy frequent itemset mining on a large set of fuzzy clusters in order to find significant patterns affecting an organism genome-wide. The tailored sensitivity of CRM predictors based on related sequences may be useful for studying the specifics of previously established mechanisms, although the application of such tools genome-wide may not be suitable due to performance restrictions and the lack of enough prior knowledge. CisMiner is, in this sense, a unique approach for obtaining reliable putative CRMs for whole-genome studies with no prior assumptions about genes or transcription factors involved.

This work is organized as follows. First, the proposed methodology is described in detail. Second, obtained results for *Saccharomyces cerevisiae* and *Drosophila melanogaster* are presented and discussed. We show that many of the obtained relations between TFs are supported by previous scientific evidence. In addition, confident new putative cis-regulatory modules have been obtained, contributing to the discovery of new regulatory relations. Finally, conclusions and future work are discussed. CisMiner is freely accessible at http://genome2.ugr.es/cisminer.

## Methods

### Methodology overview

**Figure 1:**
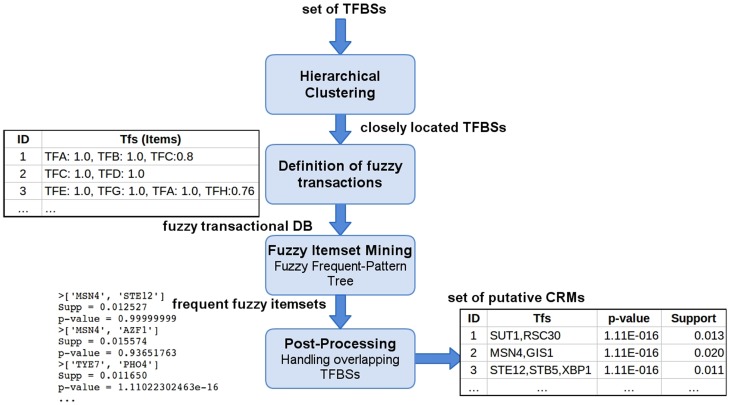
Outline of the CisMiner procedure. Diagram of the main steps of the CisMiner procedure. Given a set of TFBSs, the process starts by performing a fuzzy hierarchical clustering to obtain a set of closely located TFBSs. The result of this step is a fuzzy transactional database, which will then be mined by a Fuzzy Frequent Itemset Mining algorithm (Fuzzy Frequent-Pattern Tree) to obtain a set of frequent fuzzy itemsets. Finally, a postprocessing takes place in order to handle overlapping TFBSs that appear in each frequent itemset. As a result, a set of putative CRMs, along with their estimated p-value and their fuzzy support, is given.

CisMiner implements a data analysis pipeline that takes a set of transcription factor binding sites (TFBSs) as input and provides a set of significant co-ocurrences of any number of TFBSs as output. To this end, the following steps are performed. Given a set of TFBSs, fuzzy clusters of closely-located TFBSs are detected genome-wide. These fuzzy sets are included as itemsets in a fuzzy transactional database, which is in turn mined to obtain combinations of TFBSs which co-occur significantly. The Fuzzy Frequent-Pattern Tree (Fuzzy FP-Tree) algorithm, a fuzzy frequent itemset mining algorithm developed by the authors, is applied to this end, since it has previously shown a good performance for very large datasets [31]. The set of TFBSs used as input can either be predicted in-silico or in-vivo, allowing the user to couple this methodology to any in-silico TFBSs prediction tool to perform a prior genome-wide search for a set of putative TFBSs, which can then be given to CisMiner as an input to discover CRMs. An outline of the procedure is shown in Figure 1.

### Clustering of TFBSs and fuzzy transactional database construction

In order to be able to extract significant groups of TFs that form putative cis-regulatory modules (CRMs), the first step taken by CisMiner is to perform a fuzzy clustering of closely located TFBSs and model this set of clusters as a fuzzy transactional database.

Fuzzy set theory was proposed by Zadeh in 1965 to mathematically model the imprecision inherent to some concepts [32]. Briefly, fuzzy set theory allows an object to partially belong to a set with a membership degree between 0 and 1.

Frequent Itemset Mining was proposed by Agrawal in 1993, as an algorithm for extracting frequent itemsets from large databases [33]. Since then, a large number of algorithms have been proposed for frequent-itemset mining [34]. Given a transactional database where each transaction is a set of *items*, the aim of these techniques is to find a set of expressions of the form 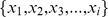, where each 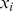 represents an item. This expression is called *itemset*. The probability that a given itemset occurs in the data base is called the *support* of the itemset. If the support of an itemset is greater than a user-specified threshold, then the itemset is said to be *frequent*. Thus, Frequent Itemset Mining algorithms aim to extract itemsets from a database with support greater than some user-specified threshold.

Frequent Itemset Mining algorithms have featured many applications that enable researchers to unveil hidden patterns in large amounts of data [35]. However, biological data are usually uncertain and imprecise. In order to be able to reflect this uncertainty, fuzzy technology has been incorporated to the Frequent Itemset Mining philosophy in the Fuzzy FP-Tree algorithm applied in this work.

Connecting fuzzy technology with frequent itemset mining allows us to incorporate uncertainty and imprecision to our knowledge model. In this sense, fuzzy itemsets are also expressions of the form 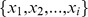, but in this case, each 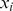 is accompanied by a value in 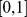 which defines its membership degree to the itemset. Fuzzy support measures the frequency of the itemset.

CisMiner combines fuzzy theory with frequent itemsets in order to increase their capacity of modelling the uncertainty present in biological data, and, in particular, TFBS binding location data. The fuzzy clusters of closely located TFBSs are modelled as itemsets in a fuzzy transactional database. In order to build such database, a group-average hierarchical clustering (the implementation of the hierarchical clustering method was obtained from the python-hcluster package release 0.2.0-1).algorithm is run over the set of TFBS locations.

An upper-threshold of 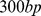 was set for stopping the cluster aggregation. That is, we seek groups of TFBSs which span around 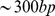 in the genome, assuming that regulatory modules are generally a few hundred base pairs in length [36].

**Figure 2:**
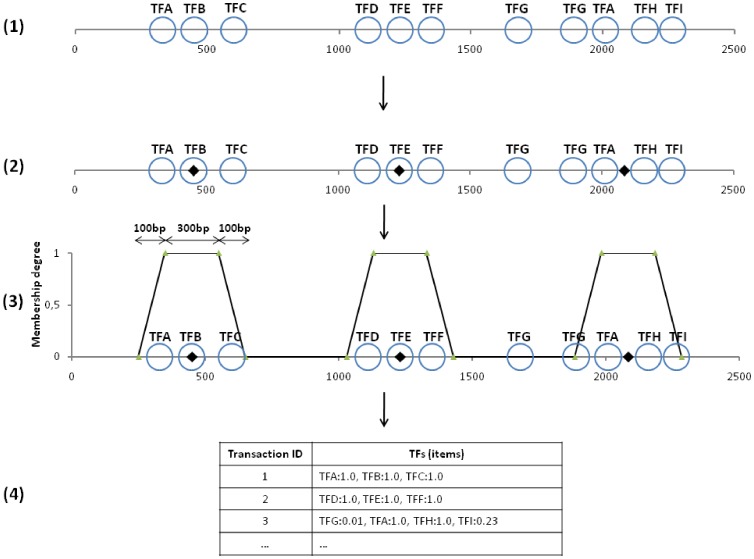
Procedure for generating the fuzzy transactional database. (1) Each circle represents a binding site. Each binding site is labeled with the name of the TF which binds that BS. (2) Three clusters are obtained. Centroids are calculated for each cluster. (3) Fuzzy sets are defined for each cluster. (4) Fuzzy transactions are generated from the fuzzy sets. The value after the colon indicates the membership degree of the corresponding TF to the transaction.

Once the list of clusters was obtained, a *fuzzy* transaction was defined for each cluster. The membership degree function for each TFBS in each cluster was defined as a trapezoidal function, as it is shown in figure 2, with the following parameters: a centroid 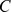 was obtained for each cluster as the median value of the position of the included TFBSs. Then, the constant region of the trapezoidal function was set from 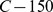 to 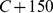. A linear increasing function in 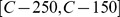 and a decreasing function in 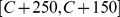 were defined to set membership degrees at the fuzzy borders of each cluster.

Once the membership degree functions were defined, a set of fuzzy transactions were built. Figure 2 shows an outline of this procedure.

### Mining the frequent fuzzy itemsets

After the fuzzy transactional database has been built, CisMiner proceeds to obtain significant sets of co-occurring TFs (i.e. putative CRMs). The fuzzy frequent itemset mining procedure enables CisMiner to remove non-significant TF clusters at a genome-wide scale. We implemented a fuzzy frequent itemset mining algorithm called the Top-Down Fuzzy Frequent Pattern-Growth algorithm, which has been developed by the authors in a previous work [31].

Briefly, this procedure works as follows (for a more exhaustive description of the Fuzzy FP-Tree data structure generation and traversing, see [31]). Initially, the algorithm scans the transaction database in order to get a sorted list of all the frequent items, i.e. items with support greater than a specified threshold. An item 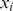 represents one TFBS and belongs to a transaction of the form 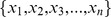 with a certain membership degree. The aim of the frequent itemset mining procedure is to extract significant itemsets, i.e. collections of items that appear frequently among the transactions in the database. After the items with higher support than the specified threshold have been selected, the items in this list along with their membership degrees for each transaction are introduced into the Fuzzy FP-Tree data structure. The efficiency of this procedure relies on the use of this tree, since it compiles all the information the algorithm needs from the transactions.

Items in each transaction that are present in the frequent items list are inserted as nodes into the Fuzzy FP-Tree according to their position in the frequent item list. If two transactions share their first frequent items they will share the same upper path to the root node.

For each item 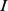, all its nodes are linked by a 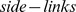 list. A vector associated to each node stores the membership degree of the transactions that belong to the corresponding item. In addition, a header table 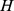 is built so each row stores the information associated to an item 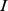: (Item, membership degrees, 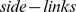). This table helps locating the nodes that correspond to each item in the Fuzzy FP-tree and to compute the fuzzy support of each itemset.

Once the Fuzzy FP-Tree and the header table 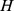 are generated, the tree is traversed in a top-down manner in order to obtain the set of frequent itemsets. Entries in 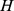 are considered one by one. For each item 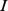 in the 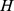 table the tree is traversed in a down-top order, starting at the nodes labeled with 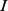. These nodes can be reached following the 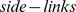 list. Each node needs a membership degree vector that keeps the minimum membership degree between the starting and the current node. This is crucial because it ensures that modifications of these vectors at upper levels do not affect the processing of lower level nodes.

Fuzzy support for each itemset was calculated as described in [37]. In addition, a p-value was calculated in order to complement the frequency value provided by the support measure. The procedure reported in [38] for the p-value computation was adapted for the fuzzy case. The null model for the calculation of this p-value represents the uninteresting situation in which no item associations are present, i.e. in which items occur independently from each other in transactions. Thus, the p-value represents the probability of the itemset to be surprising under the null-model.

### Post-processing the result set

**Figure 3:**
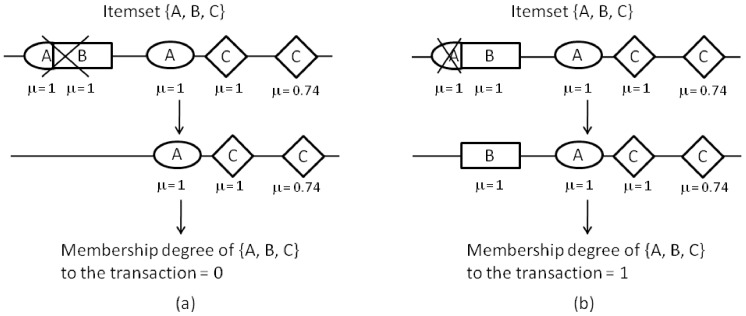
Post-processing of the results. The 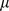 value indicates the membership degree of each binding site to its corresponding transaction. (a) Pairs of overlapping binding sites are directly removed. (b) The optimum way of fitting itemset {A, B, C} is found.

The hierarchical clustering used in the first step of the proposed methodology yields a set of closely located TFBSs. However, especially in the case where a TFBS prediction tool has been used to predict a set of TFBSs to use as input, the effects of the presence of overlapping binding sites must be considered before generating the final result set. For instance, suppose the binding sites of the transcription in Figure 3. Previous approaches directly removed both binding sites in case of overlapping [39]. This action may lead to an incorrect counting of co-occurrence, since there could be a combination of binding sites which allows the simultaneous binding of both TFs (see Figure 3.a). Hence, we look for the optimum way of fitting a given TF combination (itemset) in a given fuzzy transaction, maximizing the membership degree of the itemset to the transaction (Figure 3.b). This optimum fit is considered a putative CRM.

## Results and Discussion

We have developed an *in-silico* methodology to predict putative cis-regulatory modules (CRMs) which presents some interesting properties. First, using fuzzy sets to capture CRMs yields a more realistic model of these modules. In particular, softening their borders seems to fit the reality better than defining crisp partitions. In fact, fuzzy technology is proven to be a superior technology to enhance the interpretability of these partitions [40]. Moreover, the proposed fuzzy frequent itemset mining procedure allows to efficiently obtain any TF combination, overcoming constraints on the size and form of the recovered CRMs. Furthermore, when coupled with a TFBS prediction tool for scanning the entire non-coding genome, CisMiner does not limit the search to a set of specific regions.

The negative effects of the false positive locations generated by the TFBS search procedure are alleviated by the following filtering steps: i) Grouping the inferred TFBSs by means of a hierarchical clustering algorithm. The appearance of clusters of sites in small regions of the genome is considered a reliable indicator of regulatory function [10], [ 39], [ 41], [ 42]. ii) Searching frequent itemsets among the obtained fuzzy groups. Frequent itemset mining procedures have been successfully applied in previous approaches [39], [ 43], [ 44]. Requiring the TF combinations to repeatedly appear will help to remove spurious occurrences. iii) Calculating the statistical significance of the obtained combinations. This provides a value indicating the reliability of the results, thus allowing to remove non-relevant combinations.

In order to test the performance of CisMiner, several experiments were made. First, we used our tool to perform CRM detection from a set of validated TFBSs. In this first experiment, we avoided the uncertainty associated with the computational inference of putative TFBSs and feed CisMiner with a set of validated TFBSs in *S. cerevisiae* to test if it is able to detect biologically significant combinations. Once the good performance of the methodology was proven given a set of validated TFBSs, we tested the performance of CisMiner when considering putative TFBSs inferred by a computational tool (Patser [45], [ 46]) in *S. cerevisiae* and *D. melanogaster*. Furthermore, a comparison to the equivalent crisp technique was made. Even though the advantages of using fuzzy techniques are intuitively clear, empirical data is provided to substantiate them.

### CRM detection from validated TFBSs

This first approach was carried out over the yeast genome. More concretely, 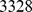 binding sites across the whole yeast genome were found for 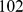 TFs in the transcription factor binding site data published by Harbison et al. [47]. With these data, 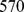 transactions were obtained with a mean of 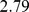 different TFs per transaction and a maximum of 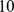 TFs in a transaction [see File S2]. CisMiner obtained 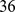 itemsets from these transactions after setting the thresholds for the support and p-value to 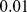. The frequency of appearance of the 96 TFs which were included in at least one transaction is provided as File S1. These frequencies indicate that there are several not very-frequent TFs in the database which are likely to generate spurious itemsets.

In order to obtain evidence supporting the found relations, STRING [48] was used. Given a set of genes, STRING looks for associations among these genes at different levels: close location in the genome, co-occurrence of the queried genes across species, individual gene fusion events, co-expression, protein-protein interactions, curated databases and text mining. STRING provided evidence of relations among all the TFs present in the itemsets returned by CisMiner for 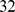 out of 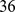 obtained itemsets, representing an 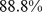 of the results. Moreover, for 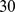 of the 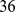 itemsets, the graphs representing the associations among their TFs are *connected* graphs, i.e. there is a path connecting each pair of TFs in the graph. The other 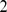 of them contained indirect relations involving only one additional transcription factor. The complete set of graphs returned by STRING for this dataset is provided as File S1.

Table 1 shows the 20 most significant TF combinations according to the computed p-value. The whole list of the 36 obtained TF combinations can also be found in File S1. STRING returned a connected graph for all combinations in Table 1 except for itemsets 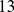 and 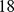. The relations among the TFs present in the predicted CRMs appear to be strong at several levels (see the corresponding graphs in supplementary material). Some of the CRMs suggested by CisMiner represent well characterised biological processes, such as the interaction of 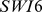 with 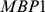 and 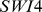 (itemset 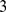) to form the MBF and SBF protein complexes, which cooperate and play a major role in progression from G1 to S phase of the cell cycle [49]. Indeed, it is worth mentioning that the combinations 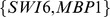 and 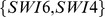 are also present at positions 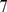 and 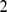, respectively. Another interesting relation is represented by itemsets 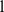, 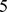 and 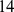, regarding transcription factors 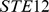, 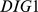, and 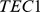. STRING returns a complete graph for these three TFs with strong empirical evindence, and a recent article by van der Felden et al. [50] also relates these three TFs. The co-ocurrence of 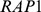 and 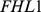 binding sites (itemset 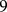) is in concordance with previous results, since both of them were shown to bind upstream of many ribosomal protein genes [51].

**Table 1:** Top-20 TF combinations.

ID	Putative CRM	p-value	Supp.	Evidence
1	STE12, DIG1	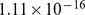	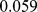	SP
2	SWI6, SWI4	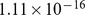	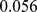	SP
3	SWI6, MBP1, SWI4	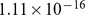	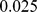	SP
4	SKN7, SOK2, PHD1	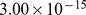	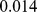	S
5	STE12, DIG1, TEC1	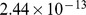	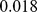	SP
6	SOK2, PHD1	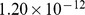	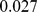	SP
7	SWI6, MBP1	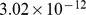	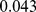	SP
8	MBP1, SWI4	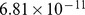	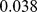	SP
9	RAP1, FHL1	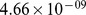	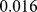	SP
10	DIG1, SWI4, TEC1	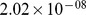	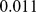	SP
11	DIG1, TEC1	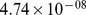	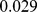	SP
12	AFT2, RCS1	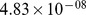	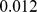	SP
13	PHD1, SUT1	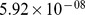	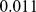	-
14	STE12, TEC1	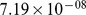	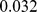	P
15	STE12, SWI6, SWI4	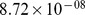	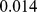	SP
16	SWI6, DIG1, SWI4	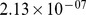	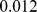	SP
17	FKH2, NDD1	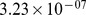	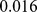	SP
18	SOK2, SUT1	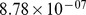	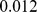	-
19	SKN7, SOK2	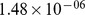	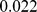	S
20	SWI6, STB1	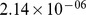	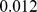	SP

First dataset. The twenty TF combinations with the lowest p-value and highest support obtained when using the dataset by Harbison et al. Evidence column shows whether results were yielded when PubMed was queried for evidence in the literature (P), STRING [48] yielded a connected graph for the given TFs (S), both conditions (SP) or none (-) were met.

No evidence was found by STRING for itemsets 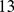 and 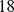. It is noteworthy to state that when queried for the single TF 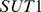, none of the genes connected to it in STRING appeared in the dataset by Harbison et al. Therefore, CisMiner was unable to find any STRING-proven relation of SUT1 to any other TF.

In addition to the STRING validation, we performed a further test using PubMed to get complementary literature-based evidences supporting the results. For the 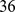 obtained CRMs, 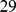 yield results when searched in PubMed, representing an 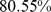 of the results. Itemsets 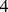, 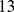, 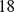 and 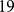 from table 1 did not yield any result when PubMed was queried. From these four itemsets, it is interesting to note that two (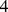 and 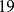) were nonetheless found as connected graphs in STRING.

Note that the lack of empirical evidence for some of the itemsets does not necessarily undermine the efectiveness of the method. Further studies and empirical evaluations are thus necessary to confirm the not-proven putative associations.

### CRM detection from putative TFBSs in *S. cerevisiae*

CisMiner has been further tested with a set of putative computationally-predicted TFBSs in the *S. cerevisiae* genome. To this aim, data were obtained from the *Saccharomyces* Genome Database (SGD) [52], [ 53] and the JASPAR database [54]. In particular, the complete yeast genome was downloaded from the SGD. JASPAR provided 177 PWMs for the yeast genome (release of January 2014).

First, the locations of potential TFBSs were inferred. For this purpose, well-known Patser and Consite tools [46], [ 55] were used, since their good performance has been repeatedly proven [39], [ 56], [ 57]. Certainly, there exist more recent TFBS detection tools which take into account positional interdependencies within a putative TFBS sequence [58]. However, their applicability is constrained by the lack of available information; these techniques require to specify the lists of sequences used to calculate each PWM, which are not usually provided by the corresponding databases. In fact, JASPAR does not provide the sequences of any of the 177 yeast motifs it contains, while TRANSFAC [59] (public release 7.0 available online) does not provide sequences for any of the 24 yeast motifs stored.

We tested the ability of both Patser and Consite to recover real TFBSs using the yeast genome and the dataset by Harbison et al. as a benchmark. We first run Patser over the complete yeast genome. Given a PWM and a sequence, Patser yields a list of values (range 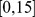) indicating how well the PWM fits each position in the sequence. Thus, we needed to select a threshold to determine which of the positions were going to be considered putative TFBSs. In addition, some TFBSs may be easier to detect than others, since each motif presents its own peculiarities. Therefore, an independent Patser-score threshold for each motif was needed [60]. We realized that it was not feasible to set Patser-score thresholds under 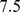. This value was selected according to the Patser documentation [61] and to our own empirical experience. It was observed that setting the thresholds under 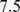 generates a huge number of putative TFBSs, thus diminishing the significance of the results. Hence, for each motif, a specific Patser-score threshold over 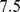 was calculated. The selection of each threshold was done so that Patser was able to detect the maximum number of TFBSs described by Harbison et al. [47].

**Figure 4:**
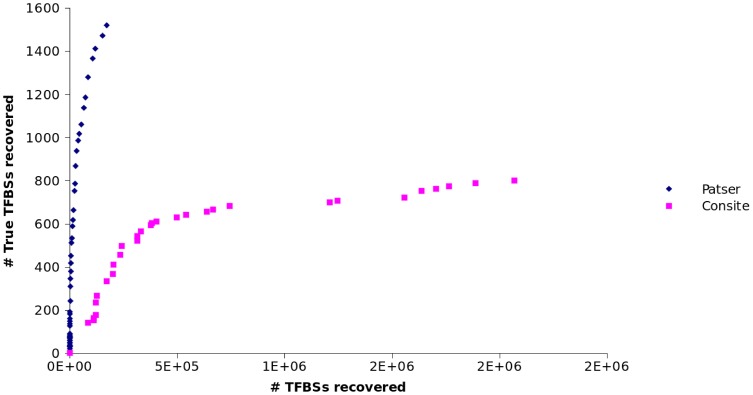
Comparison of Patser and Consite. Number of TFBSs from the dataset by Harbison et al. against the total number of TFBSs detected by Patser and Consite.

A similar procedure was carried out to test Consite's performance. In this case, for each PWM, Consite yields a list of values in the range 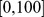. Best results were obtained for a Consite threshold below 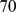. Figure 4 shows the number of *true* TFBSs recovered by each tool against the total number of sites detected. As it can be seen, Patser performs better than Consite in this particular case, since the total number of potential binding sites tends to be lower than that obtained with Consite for the same number of *true positives*. Therefore, Patser was finally selected for our purposes. The obtained thresholds for each motif are provided as File S1. Only 66 motifs are shown, since the rest of motifs were not found in the dataset by Harbison et al. We estimated the threshold for those PWMs not found in the dataset by Harbison by computing the median value of the thresholds obtained for the other 66 motifs [see File S1].

With these settings, 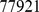 putative binding sites were detected, which include 1412 of those described by Harbison et al. These 1412 TFBSs represent the 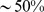 of the total number of binding sites described by these authors. It is noteworthy that, for a significant number of the motifs (e.g. ARR1, ASH1, BAS1), it was impossible to detect any of their known TFBSs, not even setting the Patser-score threshold to 0. Likewise, there were some motifs which required extremely low thresholds in order to capture their corresponding TFBSs (e.g 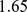, 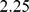, 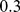 for ABF1, ADR1 & AFT2, respectively). All of this can be reflecting the biological complexity of the problem. It can also be be due to a certain incoherence between the data retrieved from JASPAR and those by Harbison et al. This could be due to the presence of outliers in both datasets or even to incoherences in the nomenclature of the motifs and TFs. The following tests are a way to show the ability of CisMiner to overcome these problems and yield some interesting results for further research from computationally inferred TFBSs. However, computational inference of TFBSs is out of the scope of this work.

Once the putative binding sites were detected, the fuzzy transactional database was built. 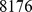 transactions were obtained with an average of 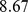 different TFs per transaction and a maximum of 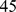 TFs in a transaction. The frequency of occurrence of each TF in the fuzzy transactional database is provided as File S1. The complete transactional data table is also provided in File S3.

CisMiner was run with the parameter settings summarized in Table 2. 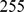 itemsets were obtained. The reported combinations of TFs showed significant p-values, indicating that many of the obtained itemsets may represent real biological associations among the corresponding transcription factors. Moreover, the obtained itemsets contain between 2 and 4 TFs, which matches size estimations by previous works [42], [ 62]. Table 3 shows a sample of the obtained itemsets. The complete set of TF combinations is also provided as File S1. It is not the aim of this paper to provide a comprehensive list and biological interpretation of all of the obtained patterns, but to show that significant associations are obtained and that many of them are in concordance with previously published knowledge. A deeper biological analysis will be the topic for future works.

**Table 2:** Parameter values.

Patser score threshold	**Motifs with known BSs**	**Rest**
	See File S1	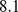
Hierarchical cluster	**Aggregation threshold**	
	300 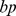	
Fuzzy TD-FP-growth	**p-value threshold**	**Support threshold**
	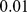	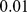

Second dataset. Summary of the input parameters used.

**Table 3:** TF combinations.

ID	Putative CRM	p-value	Support
1	STE12, TEC1	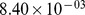	0.013
2	ADR1, RAP1	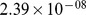	0.014
3	ADR1, RGT1	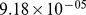	0.013
4	ADR1, SIP4	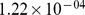	0.011
5	AFT2, RAP1	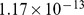	0.012
6	MIG3, RGT1	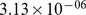	0.012
7	MSN4, RPN4	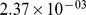	0.024
8	MSN4, SKN7	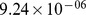	0.013
9	MSN4, GIS1	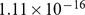	0.020
10	MIG1, MIG2	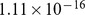	0.016
11	STE12, GCR2, STB5, XBP1	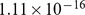	0.010
12	ADR1, MIG1	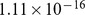	0.020
13	SUT1, MIG1	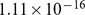	0.018

Second dataset. Some of the TF combinations obtained when using the TFBSs detected by Patser (yeast genome).

First, we wanted to check whether the methodology was able to detect the combinations obtained in Section following this second approach. Direct comparison of both result sets showed that only the itemset 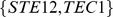 was shared. The support threshold could be filtering out the rest of the itemsets in this second approach. In fact, lowering the support threshold we were able to get up to 13 of the previous combinations. Furthermore, the TFs DIG1, NDD1, SWI6, RCS1 and STB1 were not found in JASPAR, thereby justifying the absence of 14 of the itemsets that appeared in the first result set. Finally, the rest of itemsets lost their statistical significance.

Again, STRING was used to validate the results. In this case, STRING provided evidence of direct relations among the transcription factors of 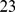 of the recovered itemsets. In addition, for the first 100 results, STRING provided direct relations between the TFs of 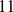 of the putative CRMs, and indirect relations (involving only 1 additional TF) for 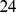. This implies that for the first 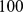 results, around 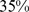 represent a direct or indirect relation among the proposed TFs. The itemset list is provided as File S1.

Here we briefly comment some of the results in Table 3. For example, STE12 and TEC1 seem strongly related (itemset 1). Both proteins are known to cooperate and regulate several cellular processes [63]–[65]. The ADR1 protein appears related to RAP1, RGT1 and SIP4 (itemsets 2–4). A number of bibliographic sources confirm such associations. ADR1 and RAP1, among other transcriptional regulators, may participate in barrier function, blocking the propagation of transcriptional silencing in yeast [66]. Likewise, the relation between ADR1 and RGT1 (YKL038W) was also found in the literature. These two factors are involved in the transcriptional response to transient perturbations in carbon source [67]. Finally, ADR1 and SIP4 participate in the transcriptional control of nonfermentative metabolism in the *Saccharomyces cerevisiae* [68].

Next itemset in Table 3 (itemset 5) contains AFT2 and RAP1, which are known to induce the expression of FRE1 in response to iron and copper depletion [53]. The next combination in Table 3 (itemset 6) involves the previously mentioned RGT1 factor, which in this case appears in cooperation with MIG3. Hazbun et al. [69] experimentally proved that both factors bind the promoter region of the gene SUC2, which product is an invertase enzyme. Then, three itemsets are shown which relate MSN4 with RPN4, SKN7 and GIS1 (itemsets 7–9), all of them representing previously described associations. Thus, MSN4, RPN4 and SKN7 are known to participate in the transcriptional response of *Saccharomyces cerevisiae* to the stress imposed by certain fungicides and herbicides [70], [ 71]. Regarding the relation between MSN4 and GIS1, STRING returned associations at different levels: experimental, curated databases and text mining. Previous authors described that the Rim15 regulon is mediated by these two transcription factors [72]. Finally, MIG1 and MIG2 also appear strongly related at different levels. Glucose repression of the SUC2 gene is dependent on MIG1 and MIG2 [73]. The last itemsets in Table 3 show some combinations for which STRING returned indirect associations, and some others for which no confirmation was obtained.

As in the previous section, a search over PubMed was performed in order to obtain more information about the complete result set. The PubMed results were manually curated and at least 23 more combinations were found to be related [see File S1].

In our aim to provide additional experimentation supporting the obtained results, the full methodology was re-run over 100 randomized yeast genomes. Interestingly, the mean number of predicted TFBSs by Patser in this random datasets was 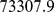, which is near the 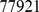 TFBSs predicted using the real dataset. Even more interestingly, only an average of 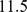 significant TF combinations remained in this case, in constrast to the 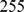 putative CRMs obtained using the real genome. This fact could be a clear evidence of the potential of the methodology to remove spurious occurrences, and suggests a false discovery rate lower than 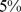.

To finish this section, it is worth mentioning that the complete process was also re-run after raising the minimum Patser-score threshold to 8. Thus, 56 itemsets were obtained (results not shown). It is interesting that this new set of combinations was a subset of the 255 itemsets analyzed in this section. Moreover, the *p*-values were almost the same to those calculated when setting the minimum Patser-score threshold to 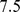. This fact suggests that the methodology is coherent and robust.

### CRM detection from putative TFBSs in *D. melanogaster*

In order to test whether the application of the methodology over larger and more complex genomes is computationally feasible and whether it can still unveil significant knowledge, a similar experiment was performed over the *Drosophila melanogaster* genome.

A comprehensive biological interpretation of the complete result set was out of the scope of this work. However, the interest of the results is discussed and verified in this section. The complete *Drosophila melanogaster* genome was downloaded from FlyBase [74], [ 75] (Release 5.56, March 2014). In addition, 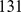 PWMs of the *Drosophila melanogaster* genome were retrieved from Jaspar. According to the Patser-score thresholds calculated above, subsequences scoring over 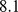 were considered as potential TFBSs. Thus, 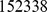 transactions were obtained with a mean of 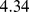 different TFs per transaction and a maximum of 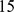 TFs in a transaction. The frequency of appearance of each TF in the fuzzy transactional database is provided as File S1. The complete transactional data table is provided as File S4.

Interestingly, only 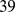 significant putative CRMs were obtained. In this case, STRING returned just one direct relation (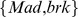, Table 4). Other 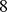 itemsets returned indirect relations when STRING was queried, representing 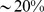 of the total obtained. Regarding PubMed searches, 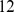 of the results returned matches when searched in PubMed. The complete list with the corresponding PubMed links is provided in File S1. However, it is extremely difficult to search for scientific evidences of the obtained relations due to the unspecific identifiers of these transcription factors, e.g.: *opa*, *btd*, *h*, *D*. The putative CRMs suggested by at least two more itemsets were found to be related by a manual inspection of the literature retrieved by PubMed. This last experiment has shown that it is computationally feasible to apply the methodology over more complex genomes. Moreover, these 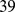 modules present significant quality values and may represent real biological interactions. Future work is needed to biologically validate and interpret all the obtained combinations.

**Table 4:** TF combinations.

Id	Putative CRM	p-value	Support
1	btd, hkb	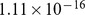	0.045
2	btd, Mad	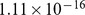	0.041
3	btd, opa	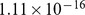	0.033
4	btd, h	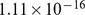	0.003
5	Mad, brk	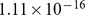	0.029
6	btd, CTCF	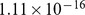	0.023
7	Mad, opa	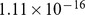	0.023
8	Mad, hkb	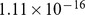	0.022
9	brk, opa	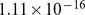	0.021
10	Mad, h	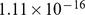	0.019

Third dataset. Some of the TF combinations obtained when using the TFBSs detected by Patser (Drosophila genome).

### Fuzzy-crisp comparison

To evaluate the contribution of fuzzy technologies to the methodology, we implemented a crisp version of the same methodology and compared the results obtained using the same quality thresholds (See Methods). The procedure to create the crisp transactional database was a crisp version of the fuzzy procedure previously described. First, a crisp clustering method was run to identify the transactions, where crisp borders were defined for each cluster at bases 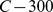 and 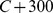 (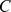 is the cluster centroid as defined in Methods). In the crisp transactional database, all items in each transaction have a membership degree of 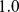. This means that a TFBS belongs to a given cluster if it is fully located within bases 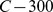 and 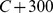. A crisp version of the frequent itemset mining algorithm was applied in this case to extract the frequent itemsets.

As expected, significant differences were found between the crisp and fuzzy results on the yeast dataset. First, the crisp algorithm obtained 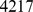 combinations while the fuzzy one returned 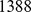, being these a subset of the crisp results [see File S5]. In order to determine whether the values of the measures obtained by the fuzzy and the crisp methodologies were significantly different, two ANOVAs were carried out (Table 5). Fuzzy sets are proven to be a superior technology to model partitions with blurry borders. Obtained results show more significant *p*-values achieved by the fuzzy methodology, suggesting that an effective prunning of spurious itemsets is achieved by applying the fuzzy algorithm.

**Table 5:** Fuzzy-crisp comparison.

	Yeast	Dmel
Mean fuzzy support	0.0084	0.0088
Mean crisp support	0.0108	0.0094
Mean fuzzy *p*-value	0.0144	0.0133
Mean crisp *p*-value	0.0070	0.0104
ANOVA support significance	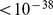	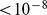
ANOVA *p*-value significance	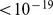	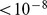

The four first rows show the mean values of fuzzy/crisp support and *p*-value of the combinations respectively. The last two rows show the statistical significance returned by the ANOVA procedure.

The same steps were taken to test fuzzy-crisp performance over the *Drosophila melanogaster* dataset. In this case, 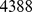 TF combinations were returned by the crisp procedure while 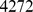 were obtained by the fuzzy one [see File S5]. Table 5 shows that the results obtained with this other dataset also comply with the previous comments. These results show the advantages of using fuzzy technology to model the TFBS clusters, improving the representation of a CRM by removing sharp borders and achieving a better performance in terms of *p*-values.

## Conclusions

In this work we have presented CisMiner, an *in silico* fuzzy methodology able to obtain putative CRMs by means of extracting significant co-occurrences of closely located TFBSs genome-wide. This methodology presents some interesting properties:

*Genome-wide scope*. CisMiner is capable of analyzing the complete non-coding genome of an organism without limiting the search space to specific regions.

*Uncertainty handling*. The uncertainty inherent to CRM detection is better modeled by fuzzy technology.

*Flexibility in TFBS number, distribution and distance*. CisMiner does not impose constraints on the form or number of elements of the discovered CRMs.

*Prior knowledge not needed*. The proposed methodology does not require any prior knowledge to restrict the search space.

*Easily interpretable results*. Each CRM is expressed as a set of TFs, its frequency of appearance in the genome and a *p*-value, which makes the results robust and easily interpretable.

*Extensible and efficient*. To the extent of our knowledge, the proposed methodology is the only one available that addresses a global genome-wide search for CRMs with no restrictions, which is efficient and robust due to the use of efficient data structures. In addition, it is easily extensible to larger genomes.

*Freely accessible*. CisMiner is available at: http://genome2.ugr.es/cisminer.

Several experiments were carried out to validate the proposed methodology. CisMiner has been shown to identify statistically significant CRMs composed of TFs with well-known interactions as reported in STRING and the literature.

The experimental results also showed that when coupled with a TFBS detection tool, the performance of the final results is strongly dependent on the performance of the TFBS detection approach.

On the other hand, although many approaches have been proposed to understand local regulatory mechanisms, little has been proposed to extract knowledge about broader regulatory mechanisms. In this sense, the genome-wide scope of CisMiner can help to shed some light in such regulatory processes.

Future work comprises testing the methodology on more complex species with larger genomes. The performed experiments have shown that the methodology is based on consistent principles and its modularity enables us to easily improve it when new methos and data are made available. In addition, we believe that the integration of additional sources of information besides sequence data (e.g. chromatin structure, protein structure) may help to refine the results.

## Supporting Information

File S1 **Complete datasets.** Additional pdf file including the full datasets obtained for both *Drosophila* and *saccharomyces*, along with the selected thresholds and STRING graphs for the first dataset.
(PDF)

File S2 **Fuzzy transactions. First dataset.** Fuzzy transactional database built when considering the dataset of real TFBSs reported by Harbison et al.
(TXT)

File S3 **Fuzzy transactions. Second dataset.** Fuzzy transactional database built when considering the TFBSs detected by Patser (*S. cerevisiae* genome).
(TXT)

File S4 **Fuzzy transactions. Third dataset.** Fuzzy transactional database built when considering the TFBSs detected by Patser (*D. melanogaster* genome).
(ZIP)

File S5 **Fuzzy and crisp comparison.** Three different tables containing the set of fuzzy itemsets used for the comparison, the set of crisp itemsets and a list with the intersection between both result sets.
(ZIP)
