# Supplementary material for: CisMiner: Genome-Wide In-Silico Cis-Regulatory Module Prediction by Fuzzy Itemset Mining
Source: PLoS One. 2014 Sep 30;9(9):e108065. doi: 10.1371/journal.pone.0108065 (PMC4182448; doi:10.1371/journal.pone.0108065)
Supplement: File S1 — Complete datasets. Additional pdf file including the full datasets obtained for both Drosophila and saccharomyces, along with the selected thresholds and STRING graphs for the first dataset. (PDF) [file pone.0108065.s001.pdf]

Supplementary material.

June 9, 2014

1 Frequency of TFs. First dataset

Frequency of appearance of the TFs in the database obtained using the dataset of real TFBSs reported by Harbison et al.

Figure 1: Frequency of appearance of each TF in the transactions databaseusing the dataset of real TFBSs reported by Harbison et al. Only TFs that appear at least once are shown.

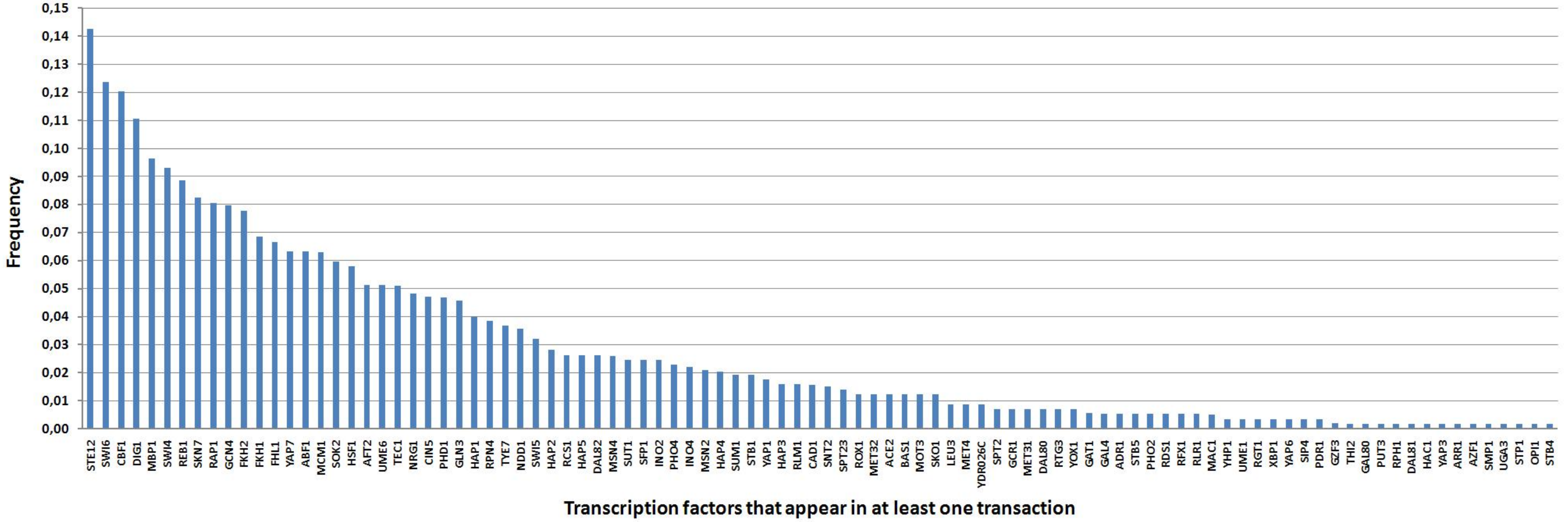

2 Complete result set. First dataset.

Complete set of putative CRMs obtained using the dataset of real TFBSs reported by Harbison et al. Each combination appears linked to its corresponding PubMed result list if it yields any literature evidence. For each putative CRM, the graph returned by STRING is shown. The meaning of the edge color is explained in figure 2

Table 1: Significant CRMs in the dataset by Harbison et al.

#	Transcription Factors	p-value	Support	STRING graph
1	*STE12, DIG1*	1.11×10^{-16}	0.059	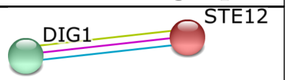
2	*SWI6, SWI4*	1.11×10^{-16}	0.056	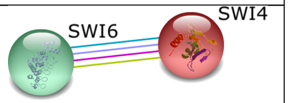
3	*SWI6, MBP1, SWI4*	1.11×10^{-16}	0.025	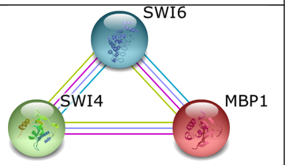
4	*SKN7, SOK2, PHD1*	3.00×10^{-15}	0.014	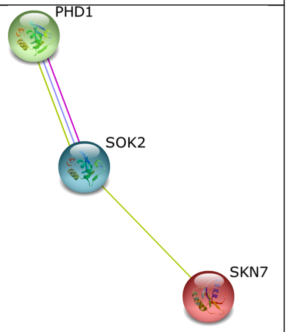
5	*STE12, DIG1, TEC1*	2.44×10^{-13}	0.018	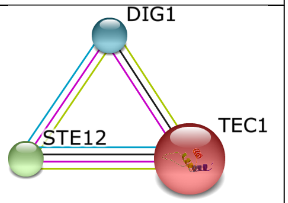
6	*SOK2, PHD1*	1.20×10^{-12}	0.027	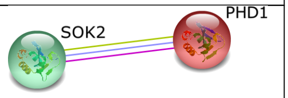
7	*SWI6, MBP1*	3.02×10^{-12}	0.043	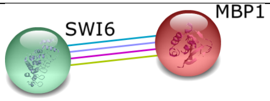

8	$MBP1, SWI4$	6.81×10^{-11}	0.038	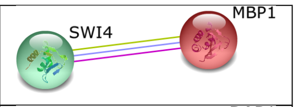
9	$RAP1, FHL1$	4.66×10^{-09}	0.016	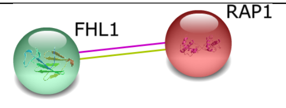
10	$DIG1, SWI4, TEC1$	2.02×10^{-08}	0.011	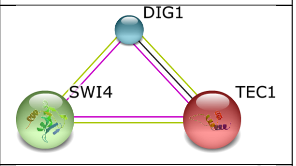
11	$DIG1, TEC1$	4.74×10^{-08}	0.029	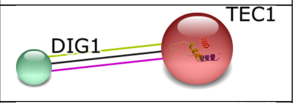
12	$AFT2, RCS1$	4.83×10^{-08}	0.012	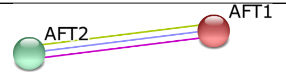
13	$PHD1, SUT1$	5.92×10^{-08}	0.011	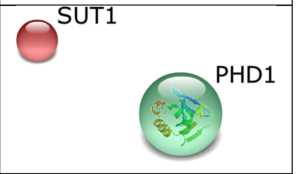
14	$STE12, TEC1$	7.19×10^{-08}	0.032	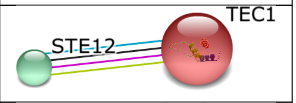
15	$STE12, SWI6, SWI4$	8.72×10^{-08}	0.014	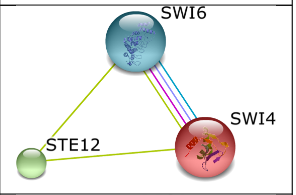
16	$SWI6, DIG1, SWI4$	2.13×10^{-07}	0.012	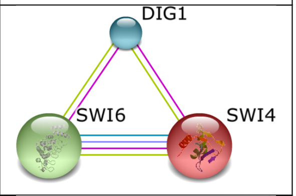
17	$FKH2, NDD1$	3.23×10^{-07}	0.016	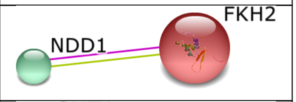
18	$SOK2, SUT1$	8.78×10^{-07}	0.012	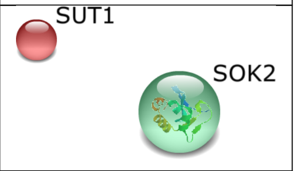
19	$SKN7, SOK2$	1.48×10^{-06}	0.022	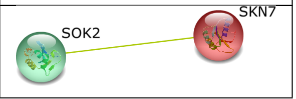

20	$SWI6, STB1$	2.14×10^{-06}	0.012	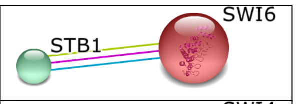
21	$SWI4, STB1$	2.93×10^{-06}	0.011	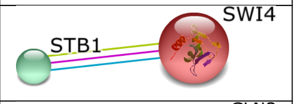
22	$GLN3, DAL82$	3.72×10^{-06}	0.011	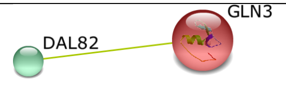
23	$FKH2, FKH1$	2.55×10^{-05}	0.015	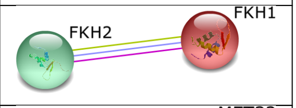
24	$CBF1, MET32$	2.79×10^{-05}	0.011	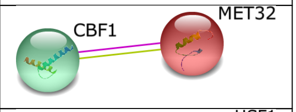
25	$HSF1, MSN4$	3.16×10^{-05}	0.011	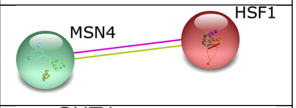
26	$SKN7, SUT1$	3.59×10^{-05}	0.011	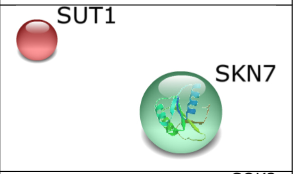
27	$SOK2, CIN5$	4.51×10^{-05}	0.015	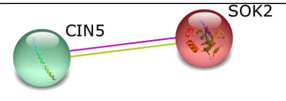
28	$SWI4, TEC1$	1.13×10^{-04}	0.018	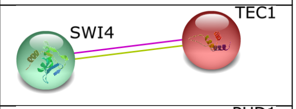
29	$SKN7, PHD1$	1.61×10^{-04}	0.015	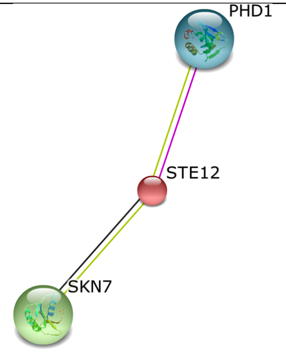
30	$FKH2, MCM1$	6.30×10^{-04}	0.014	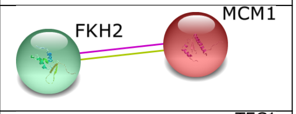
31	$SWI6, TEC1$	1.18×10^{-03}	0.018	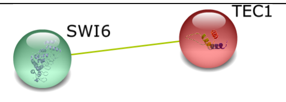

32	$GCN4, GLN3$	1.34×10^{-03}	0.012	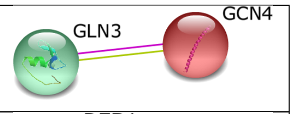
33	$REB1, RPN4$	3.88×10^{-03}	0.011	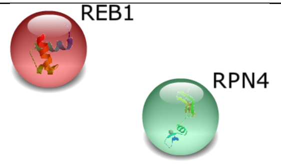
34	$SWI6, FKH2$	4.07×10^{-03}	0.021	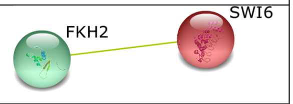
35	$SKN7, NRG1$	6.09×10^{-03}	0.010	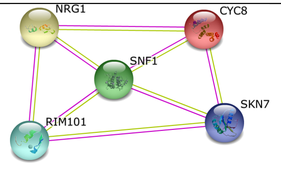
36	$DIG1, SWI4$	7.03×10^{-03}	0.021	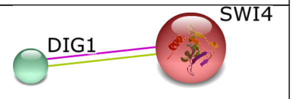

Figure 2: Meaning of edge color in STRING graphs.

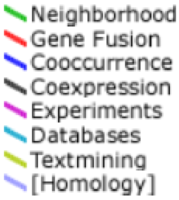

3 Obtained thresholds for each motif

Patser-score thresholds calculated for each motif.

Transcription Factor	Computed Threshold
DAL81	15.00
ZAP1	14.85
PDR3	12.60
YDR026C	11.40
SNT2	11.25
LEU3	10.05
BAS1	9.90
RPH1	9.90
RDS1	9.75
MET31	9.60
GAL4	9.45
OPI1	9.30
CAD1	9.30
RIM101	9.30
HAP3	9.15
HAP5	9.00
STP1	8.85
INO4	8.85
FKH2	8.85
RTG3	8.70
AZF1	8.70
RLM1	8.70
HAP1	8.55
SWI5	8.40
CBF1	8.40
MET4	8.40
RFX1	8.40
UME6	8.25
PHO4	8.25
PUT3	8.25
ROX1	8.25
RPN4	8.25
TYE7	8.10
GCR1	8.10
INO2	8.10
ACE2	8.10

SKN7	7.95
TEC1	7.95
STB4	7.95
STE12	7.95
GAT1	7.95
SIP4	7.80
SWI4	7.80
SPT23	7.80
HAP4	7.80
MSN4	7.80
REB1	7.80
CIN5	7.80
MET32	7.80
ABF1	7.80
SOK2	7.65
SUT1	7.65
THI2	7.65
STB5	7.65
HSF1	7.65
NRG1	7.65
PHD1	7.65
RAP1	7.65
MAC1	7.65
MBP1	7.65
MCM1	7.65
ADR1	7.65
AFT2	7.65
FKH1	7.65
YAP7	7.65
RGT1	7.65

4 Threshold selection for those JASPAR motifs which were not included in the dataset by Harbison et al.

As commented in the manuscript, for some of the JASPAR motifs we could not find a reference set of real TFBSs. In an attempt to select the most appropriate Patser-score threshold in these cases, the already calculated thresholds for the rest of Jaspar motifs were analyzed. The aim of this analysis was to find some type of dependence between the threshold-value and certain motif features, such as its length, its *information content* or the *information content* of its core. Finding such dependencies could had helped to set an appropriate threshold for these motifs according to their particular properties.

The motif length is calculated as the length of the TFBSs it represents. For example, the length of the following motif is 10:

AATAACGGAA
AATAACGGAA
CATAACGGAA
GCTAACGGCA
TGAAACTTGG
TACAACTGAA

The *information content* of a given position in the motif represents the conservation level of that position. In other words, it indicates whether certain base(s) appear clearly in that position, or the four bases may appear in that position. For a given position, the *information content* is calculated as:

$$2 + \sum_{\beta \in \{A,C,G,T\}} W_\beta log_2(W_\beta),$$

where W_β represents the relative frequency of appearance of base β in that position. For example, the information content of the first position of the previous motif may be calculated as:

$$2 + \frac{2}{10} \cdot log_2\left(\frac{2}{10}\right) + \frac{1}{10} \cdot log_2\left(\frac{1}{10}\right) + 2 + \frac{1}{10} \cdot log_2\left(\frac{1}{10}\right) + \frac{2}{10} \cdot log_2\left(\frac{2}{10}\right) =$$
$$= 2 + (-0.46) + (-0.33) + (-0.33) + (-0.46) = 0.42$$

The *core* of a motif is formed by its 5 most-conserved and consecutive positions, i.e. the 5 consecutive positions with the highest information content.

Thus, we looked for dependencies between the motif length, the information content of the motif, the core information content and the already calculated thresholds. In order to do this, the threshold values were plotted against these three properties (Figure 3). As can be seen in Figure 3, no clear trend was observed which could allow us to infer a threshold value depending on any of these features. Some other motif features may be studied in future works to see whether any interesting feature may strongly determine the ability to detect the motif occurrences in a sequence.

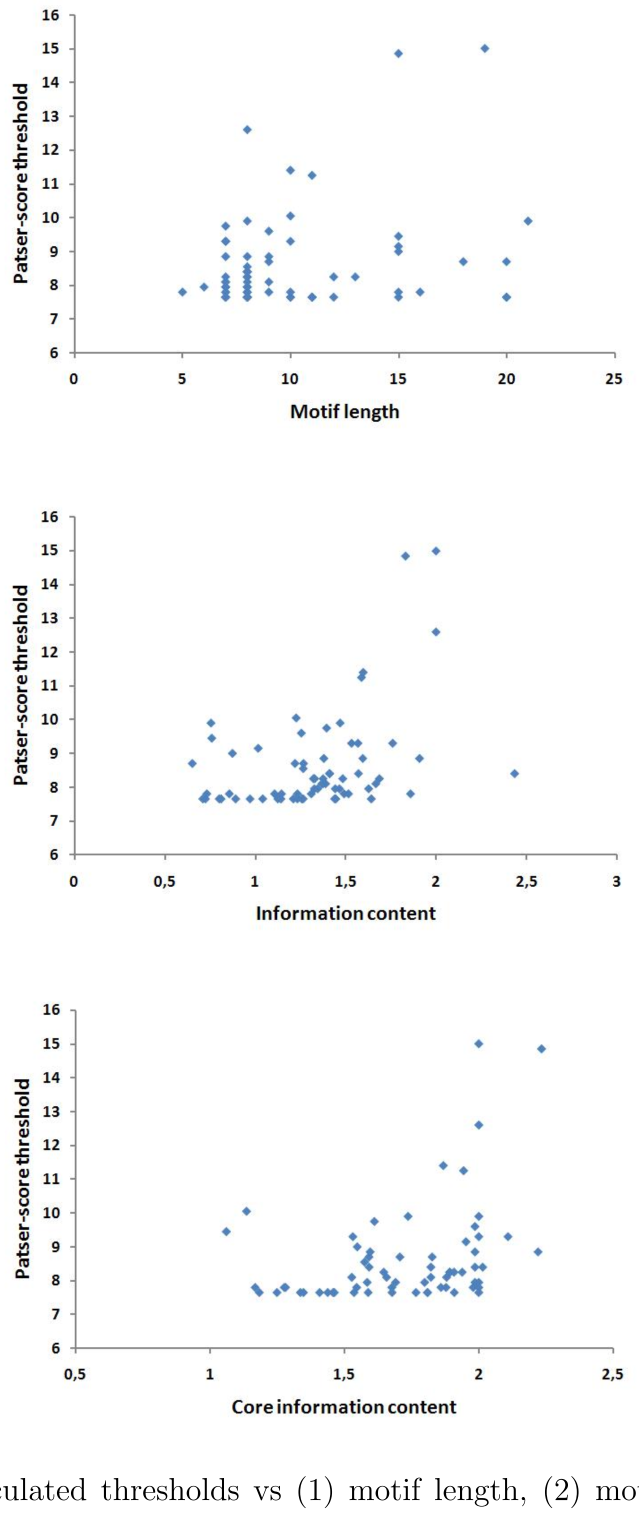

Figure 3: Calculated thresholds vs (1) motif length, (2) motif IC and (3) core IC.

4.1 Frequency of TFs. Second dataset.

Frequency of appearance of the TFs in the database obtained when using the TFBSs detected by Patser (yeast genome).

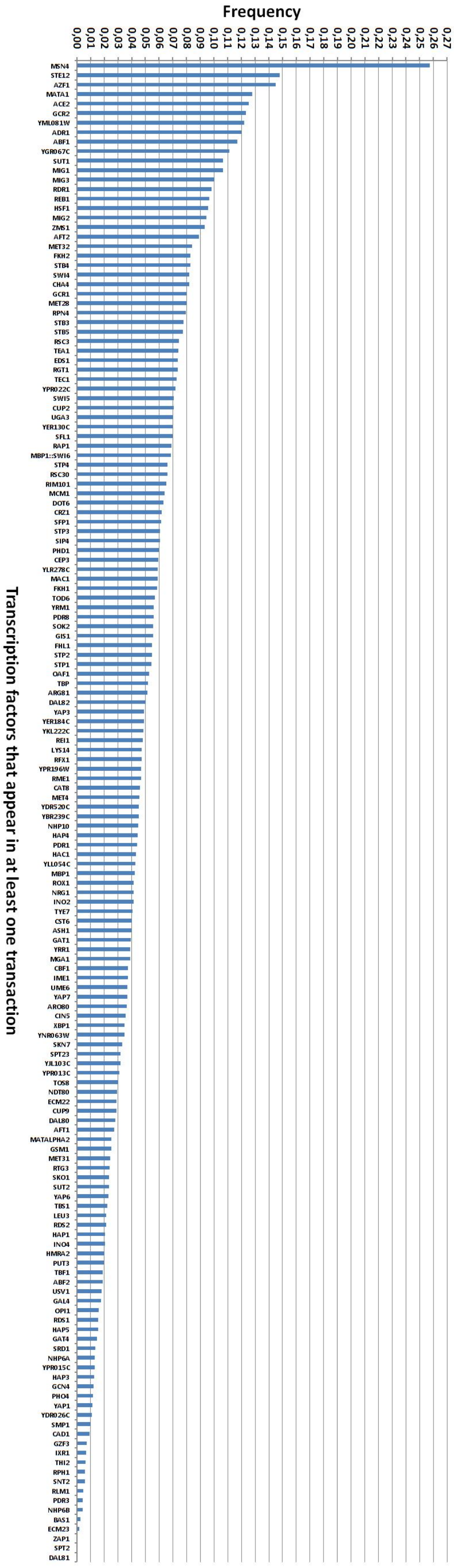
Frequency
Transcription factors that appear in at least one transaction

4.2 Complete result set. Second dataset.

Complete set of the combinations obtained when using the TFBSs detected by Patser. Each combination appears linked to its corresponding PubMed search if it yields some literature evidence (yeast genome).

#	Transcription factors	p-value	Support
1	$MSN4, YML081W$	1.11×10^{-16}	0.041
2	$MSN4, YGR067C$	1.11×10^{-16}	0.039
3	$STE12, GCR2$	1.11×10^{-16}	0.033
4	$MSN4, ZMS1$	1.11×10^{-16}	0.029
5	$GCR2, STB5$	1.11×10^{-16}	0.023
6	$MSN4, YER130C$	1.11×10^{-16}	0.022
7	$STE12, STB5$	1.11×10^{-16}	0.022
8	$YML081W, ADR1$	1.11×10^{-16}	0.021
9	$ADR1, YGR067C$	1.11×10^{-16}	0.020
10	$ADR1, SUT1$	1.11×10^{-16}	0.020
11	$YML081W, MIG1$	1.11×10^{-16}	0.020
12	$ADR1, MIG1$	1.11×10^{-16}	0.020
13	$MSN4, GIS1$	1.11×10^{-16}	0.020
14	$YML081W, SUT1$	1.11×10^{-16}	0.019
15	$YML081W, MIG3$	1.11×10^{-16}	0.019
16	$ADR1, MIG3$	1.11×10^{-16}	0.019
17	$GCR1, STB5$	1.11×10^{-16}	0.019
18	$YGR067C, SUT1$	1.11×10^{-16}	0.018
19	$YGR067C, MIG1$	1.11×10^{-16}	0.018
20	$SUT1, MIG1$	1.11×10^{-16}	0.018
21	$YGR067C, MIG3$	1.11×10^{-16}	0.018
22	$MSN4, REI1$	1.11×10^{-16}	0.017
23	$YML081W, MIG2$	1.11×10^{-16}	0.017
24	$ADR1, MIG2$	1.11×10^{-16}	0.017
25	$SUT1, MIG3$	1.11×10^{-16}	0.017
26	$STE12, XBP1$	1.11×10^{-16}	0.016
27	$GCR2, XBP1$	1.11×10^{-16}	0.016
28	$YGR067C, MIG2$	1.11×10^{-16}	0.016
29	$SUT1, MIG2$	1.11×10^{-16}	0.016
30	$MIG1, MIG2$	1.11×10^{-16}	0.016
31	$YML081W, YGR067C$	1.11×10^{-16}	0.016
32	$MIG1, MIG3$	1.11×10^{-16}	0.016
33	$GCR2, FHL1$	1.11×10^{-16}	0.016

34	$STE12, GCR2, STB5$	1.11×10^{-16}	0.016
35	$ADR1, ZMS1$	1.11×10^{-16}	0.015
36	$SUT1, ZMS1$	1.11×10^{-16}	0.015
37	$MIG1, ZMS1$	1.11×10^{-16}	0.015
38	$GCR1, XBP1$	1.11×10^{-16}	0.014
39	$MIG3, MIG2$	1.11×10^{-16}	0.014
40	$YGR067C, YPR022C$	1.11×10^{-16}	0.014
41	$MIG1, YPR022C$	1.11×10^{-16}	0.014
42	$SUT1, RSC3$	1.11×10^{-16}	0.013
43	$STB5, XBP1$	1.11×10^{-16}	0.013
44	$YML081W, YPR022C$	1.11×10^{-16}	0.013
45	$ADR1, YPR022C$	1.11×10^{-16}	0.013
46	$MIG3, YPR022C$	1.11×10^{-16}	0.013
47	$YML081W, ZMS1$	1.11×10^{-16}	0.013
48	$YGR067C, ZMS1$	1.11×10^{-16}	0.013
49	$MIG3, ZMS1$	1.11×10^{-16}	0.013
50	$MIG2, ZMS1$	1.11×10^{-16}	0.013
51	$STE12, GCR2, XBP1$	1.11×10^{-16}	0.012
52	$MIG2, YPR022C$	1.11×10^{-16}	0.012
53	$GCR1, FHL1$	1.11×10^{-16}	0.012
54	$STB5, FHL1$	1.11×10^{-16}	0.012
55	$SUT1, RSC30$	1.11×10^{-16}	0.012
56	$STE12, STB5, XBP1$	1.11×10^{-16}	0.011
57	$GCR2, STB5, XBP1$	1.11×10^{-16}	0.011
58	$GCR1, STB5, XBP1$	1.11×10^{-16}	0.011
59	$STE12, GCR2, STB5, XBP1$	1.11×10^{-16}	0.010
60	$SUT1, CHA4$	4.44×10^{-16}	0.013
61	$UGA3, RSC30$	1.22×10^{-15}	0.011
62	$MIG3, SIP4$	2.44×10^{-15}	0.010
63	$MIG3, CHA4$	7.88×10^{-14}	0.013
64	$AFT2, RAP1$	1.17×10^{-13}	0.012
65	$MIG1, CHA4$	2.46×10^{-13}	0.013
66	$MIG2, CHA4$	3.87×10^{-13}	0.012
67	$YGR067C, CRZ1$	8.90×10^{-13}	0.013
68	$MIG3, STP2$	1.36×10^{-12}	0.011
69	$MIG3, RSC3$	1.83×10^{-12}	0.013
70	$SUT1, STP2$	3.22×10^{-12}	0.010
71	$YGR067C, RSC30$	4.00×10^{-12}	0.013

72	$MIG2, REI1$	4.15×10^{-12}	0.010
73	$MIG3, CRZ1$	4.24×10^{-12}	0.012
74	$SUT1, UGA3$	4.49×10^{-12}	0.012
75	$MIG3, RSC30$	5.94×10^{-12}	0.012
76	$MIG1, CRZ1$	6.82×10^{-12}	0.012
77	$RSC3, UGA3$	1.01×10^{-11}	0.011
78	$MIG1, SIP4$	2.25×10^{-11}	0.011
79	$MIG2, CRZ1$	2.61×10^{-11}	0.011
80	$RDR1, CHA4$	3.49×10^{-11}	0.011
81	$MSN4, MIG2$	3.55×10^{-11}	0.034
82	$MIG1, REI1$	7.75×10^{-11}	0.011
83	$YGR067C, REI1$	9.04×10^{-11}	0.011
84	$ADR1, CRZ1$	1.03×10^{-10}	0.013
85	$MIG2, STP2$	1.35×10^{-10}	0.010
86	$YGR067C, STP2$	1.54×10^{-10}	0.011
87	$MIG2, RSC3$	1.82×10^{-10}	0.011
88	$MIG3, REI1$	1.86×10^{-10}	0.010
89	$MIG1, RSC30$	2.11×10^{-10}	0.012
90	$YGR067C, RSC3$	2.71×10^{-10}	0.014
91	$MIG1, RSC3$	3.53×10^{-10}	0.013
92	$ZMS1, CHA4$	7.24×10^{-10}	0.011
93	$SUT1, MCM1$	9.36×10^{-10}	0.013
94	$ADR1, STP2$	1.24×10^{-09}	0.012
95	$YML081W, RSC30$	1.93×10^{-09}	0.014
96	$YML081W, CRZ1$	1.93×10^{-09}	0.012
97	$MIG1, STP2$	2.32×10^{-09}	0.011
98	$MSN4, MIG3$	2.70×10^{-09}	0.035
99	$MSN4, CRZ1$	3.12×10^{-09}	0.022
100	$MIG2, RSC30$	4.22×10^{-09}	0.011
101	$SUT1, RPN4$	8.00×10^{-09}	0.014
102	$ADR1, REI1$	9.78×10^{-09}	0.011
103	$YGR067C, CHA4$	1.47×10^{-08}	0.012
104	$YML081W, REI1$	1.53×10^{-08}	0.011
105	$MIG3, RDR1$	1.59×10^{-08}	0.016
106	$RDR1, MIG2$	2.37×10^{-08}	0.015
107	$ADR1, RAP1$	2.39×10^{-08}	0.014
108	$ADR1, YPR196W$	3.12×10^{-08}	0.010
109	$YGR067C, UGA3$	3.79×10^{-08}	0.013

110	$MSN4, MIG1$	4.06×10^{-08}	0.037
111	$MIG2, SIP4$	4.38×10^{-08}	0.010
112	$ADR1, UGA3$	4.81×10^{-08}	0.014
113	$ZMS1, RSC30$	5.27×10^{-08}	0.010
114	$YML081W, AFT2$	6.24×10^{-08}	0.016
115	$MIG1, UGA3$	1.39×10^{-07}	0.012
116	$MIG1, RDR1$	1.82×10^{-07}	0.016
117	$STE12, FHL1$	2.10×10^{-07}	0.013
118	$YML081W, CHA4$	2.19×10^{-07}	0.012
119	$ADR1, CHA4$	3.48×10^{-07}	0.013
120	$MSN4, ADR1$	3.68×10^{-07}	0.039
121	$ZMS1, RSC3$	4.03×10^{-07}	0.010
122	$ADR1, RSC30$	4.15×10^{-07}	0.012
123	$ADR1, RSC3$	4.42×10^{-07}	0.013
124	$MIG3, UGA3$	5.67×10^{-07}	0.011
125	$YGR067C, RDR1$	6.49×10^{-07}	0.016
126	$MSN4, YPR022C$	7.96×10^{-07}	0.025
127	$MIG2, UGA3$	7.98×10^{-07}	0.011
128	$ABF1, DOT6$	9.36×10^{-07}	0.012
129	$YML081W, STP2$	1.12×10^{-06}	0.011
130	$MIG1, YLR278C$	1.32×10^{-06}	0.011
131	$SUT1, RDR1$	1.44×10^{-06}	0.015
132	$MIG1, RGT1$	1.62×10^{-06}	0.012
133	$ADR1, RDR1$	1.77×10^{-06}	0.017
134	$SUT1, YPR022C$	1.83×10^{-06}	0.012
135	$MIG3, AFT2$	2.06×10^{-06}	0.013
136	$YML081W, RSC3$	2.16×10^{-06}	0.014
137	$MSN4, SUT1$	2.26×10^{-06}	0.035
138	$ABF1, TOD6$	2.95×10^{-06}	0.011
139	$YGR067C, AFT2$	3.01×10^{-06}	0.014
140	$MIG3, RGT1$	3.13×10^{-06}	0.012
141	$YML081W, RGT1$	3.42×10^{-06}	0.014
142	$YGR067C, SIP4$	3.48×10^{-06}	0.011
143	$YGR067C, EDS1$	4.05×10^{-06}	0.013
144	$YGR067C, RAP1$	4.90×10^{-06}	0.012
145	$SUT1, SIP4$	5.08×10^{-06}	0.010
146	$MSN4, RSC30$	7.30×10^{-06}	0.023
147	$YGR067C, RGT1$	7.83×10^{-06}	0.013

148	$MSN4, SKN7$	9.24×10^{-06}	0.013
149	$YML081W, EDS1$	9.37×10^{-06}	0.013
150	$YML081W, RPN4$	1.11×10^{-05}	0.014
151	$SUT1, TEA1$	1.29×10^{-05}	0.010
152	$MSN4, UGA3$	1.44×10^{-05}	0.024
153	$STE12, GCR1$	1.52×10^{-05}	0.014
154	$ABF1, UGA3$	1.56×10^{-05}	0.012
155	$ADR1, STP1$	1.61×10^{-05}	0.010
156	$SUT1, RGT1$	1.89×10^{-05}	0.012
157	$MSN4, YLL054C$	2.00×10^{-05}	0.016
158	$ZMS1, RGT1$	2.03×10^{-05}	0.011
159	$MIG3, TEA1$	2.04×10^{-05}	0.010
160	$RDR1, RAP1$	2.09×10^{-05}	0.011
161	$ADR1, MCM1$	2.61×10^{-05}	0.011
162	$MIG2, EDS1$	3.02×10^{-05}	0.011
163	$RDR1, ZMS1$	3.11×10^{-05}	0.013
164	$YML081W, UGA3$	4.24×10^{-05}	0.012
165	$ADR1, MET28$	4.47×10^{-05}	0.014
166	$YML081W, MET28$	4.62×10^{-05}	0.014
167	$MATA1, STB5$	4.66×10^{-05}	0.014
168	$YML081W, SIP4$	4.85×10^{-05}	0.011
169	$SUT1, MET32$	5.07×10^{-05}	0.013
170	$MIG2, RGT1$	6.58×10^{-05}	0.011
171	$RDR1, RSC3$	8.45×10^{-05}	0.010
172	$ADR1, RGT1$	9.18×10^{-05}	0.013
173	$ADR1, AFT2$	9.22×10^{-05}	0.013
174	$ADR1, SIP4$	1.22×10^{-04}	0.011
175	$MIG1, EDS1$	1.24×10^{-04}	0.012
176	$ABF1, MIG3$	1.39×10^{-04}	0.016
177	$MIG3, EDS1$	1.55×10^{-04}	0.011
178	$ZMS1, EDS1$	1.64×10^{-04}	0.010
179	$MSN4, STP2$	1.65×10^{-04}	0.018
180	$MIG2, RPN4$	1.71×10^{-04}	0.011
181	$AFT2, MET28$	1.72×10^{-04}	0.011
182	$MIG1, RAP1$	1.98×10^{-04}	0.010
183	$MIG3, RPN4$	2.03×10^{-04}	0.011
184	$YML081W, RAP1$	2.05×10^{-04}	0.012
185	$ABF1, STB3$	2.34×10^{-04}	0.013

186	$SUT1, EDS1$	2.62×10^{-04}	0.011
187	$MIG2, MET28$	2.65×10^{-04}	0.011
188	$YGR067C, YER130C$	2.82×10^{-04}	0.011
189	$ACE2, MET28$	3.10×10^{-04}	0.014
190	$GCR2, STB4$	3.20×10^{-04}	0.014
191	$YML081W, RDR1$	3.49×10^{-04}	0.016
192	$ACE2, MET32$	3.50×10^{-04}	0.014
193	$SUT1, RAP1$	3.68×10^{-04}	0.010
194	$MSN4, RSC3$	4.00×10^{-04}	0.024
195	$MSN4, HAP4$	4.36×10^{-04}	0.015
196	$MATA1, GCR1$	4.93×10^{-04}	0.014
197	$MIG2, AFT2$	5.00×10^{-04}	0.011
198	$ADR1, EDS1$	5.13×10^{-04}	0.012
199	$MSN4, PDR1$	5.47×10^{-04}	0.015
200	$MATA1, FHL1$	5.53×10^{-04}	0.010
201	$ADR1, YLR278C$	5.60×10^{-04}	0.010
202	$ZMS1, RPN4$	5.75×10^{-04}	0.010
203	$MIG3, MET28$	5.75×10^{-04}	0.011
204	$MIG1, TEA1$	6.34×10^{-04}	0.010
205	$SUT1, STB4$	6.78×10^{-04}	0.012
206	$ABF1, MIG1$	7.51×10^{-04}	0.016
207	$MSN4, NRG1$	7.58×10^{-04}	0.013
208	$MIG1, MET28$	7.77×10^{-04}	0.011
209	$YGR067C, RPN4$	8.42×10^{-04}	0.011
210	$YGR067C, MET28$	8.53×10^{-04}	0.012
211	$MIG1, AFT2$	9.07×10^{-04}	0.012
212	$MSN4, EDS1$	9.41×10^{-04}	0.023
213	$MIG1, YER130C$	1.00×10^{-03}	0.010
214	$MSN4, STP1$	1.04×10^{-03}	0.018
215	$SUT1, AFT2$	1.07×10^{-03}	0.012
216	$RDR1, RPN4$	1.20×10^{-03}	0.011
217	$ADR1, RPN4$	1.45×10^{-03}	0.012
218	$MSN4, RGT1$	1.63×10^{-03}	0.023
219	$MSN4, PHD1$	1.82×10^{-03}	0.019
220	$MIG2, MET32$	1.86×10^{-03}	0.011
221	$MSN4, AFT2$	1.86×10^{-03}	0.025
222	$ACE2, RSC30$	2.24×10^{-03}	0.011
223	$MSN4, RPN4$	2.37×10^{-03}	0.024

224	$MIG1, RPN4$	2.38×10^{-03}	0.011
225	$YGR067C, TEA1$	2.62×10^{-03}	0.011
226	$ACE2, UGA3$	3.04×10^{-03}	0.011
227	$AZF1, SFL1$	3.04×10^{-03}	0.013
228	$MSN4, YPR196W$	3.37×10^{-03}	0.015
229	$AZF1, STB3$	3.65×10^{-03}	0.014
230	$ADR1, TEA1$	3.73×10^{-03}	0.012
231	$MIG3, MET32$	3.75×10^{-03}	0.011
232	$ADR1, YER130C$	3.76×10^{-03}	0.011
233	$ABF1, SUT1$	3.97×10^{-03}	0.015
234	$ADR1, MET32$	4.20×10^{-03}	0.013
235	$RDR1, SWI4$	4.30×10^{-03}	0.010
236	$ZMS1, AFT2$	4.64×10^{-03}	0.010
237	$ACE2, MCM1$	4.71×10^{-03}	0.011
238	$YML081W, YER130C$	4.77×10^{-03}	0.011
239	$MSN4, NHP10$	4.91×10^{-03}	0.014
240	$AZF1, DOT6$	4.97×10^{-03}	0.012
241	$MSN4, MET32$	5.02×10^{-03}	0.025
242	$MATA1, GCR2$	6.42×10^{-03}	0.019
243	$MSN4, ASH1$	7.08×10^{-03}	0.013
244	$MSN4, STP4$	8.34×10^{-03}	0.020
245	$MIG2, STB4$	8.40×10^{-03}	0.010
246	$STE12, TEC1$	8.40×10^{-03}	0.013
247	$ACE2, RSC3$	8.43×10^{-03}	0.012
248	$ABF1, MIG2$	8.45×10^{-03}	0.013
249	$MSN4, IME1$	8.46×10^{-03}	0.012
250	$ABF1, CHA4$	8.91×10^{-03}	0.012
251	$YML081W, MET32$	9.17×10^{-03}	0.013
252	$MIG1, MET32$	9.41×10^{-03}	0.011
253	$MSN4, CHA4$	9.54×10^{-03}	0.024
254	$AZF1, EDS1$	9.57×10^{-03}	0.013
255	$SUT1, MET28$	9.85×10^{-03}	0.011

4.3 Frequencies of TFs. Third dataset.

Frequency of appearance of the TFs in the database obtained when using the TFBSs detected by Patser (Drosophila genome).

Figure 4: Frequency of appearance of each TF in the transactions database for the execution of patser over the Drosophila genome. Only TFs that appear at least once are shown.

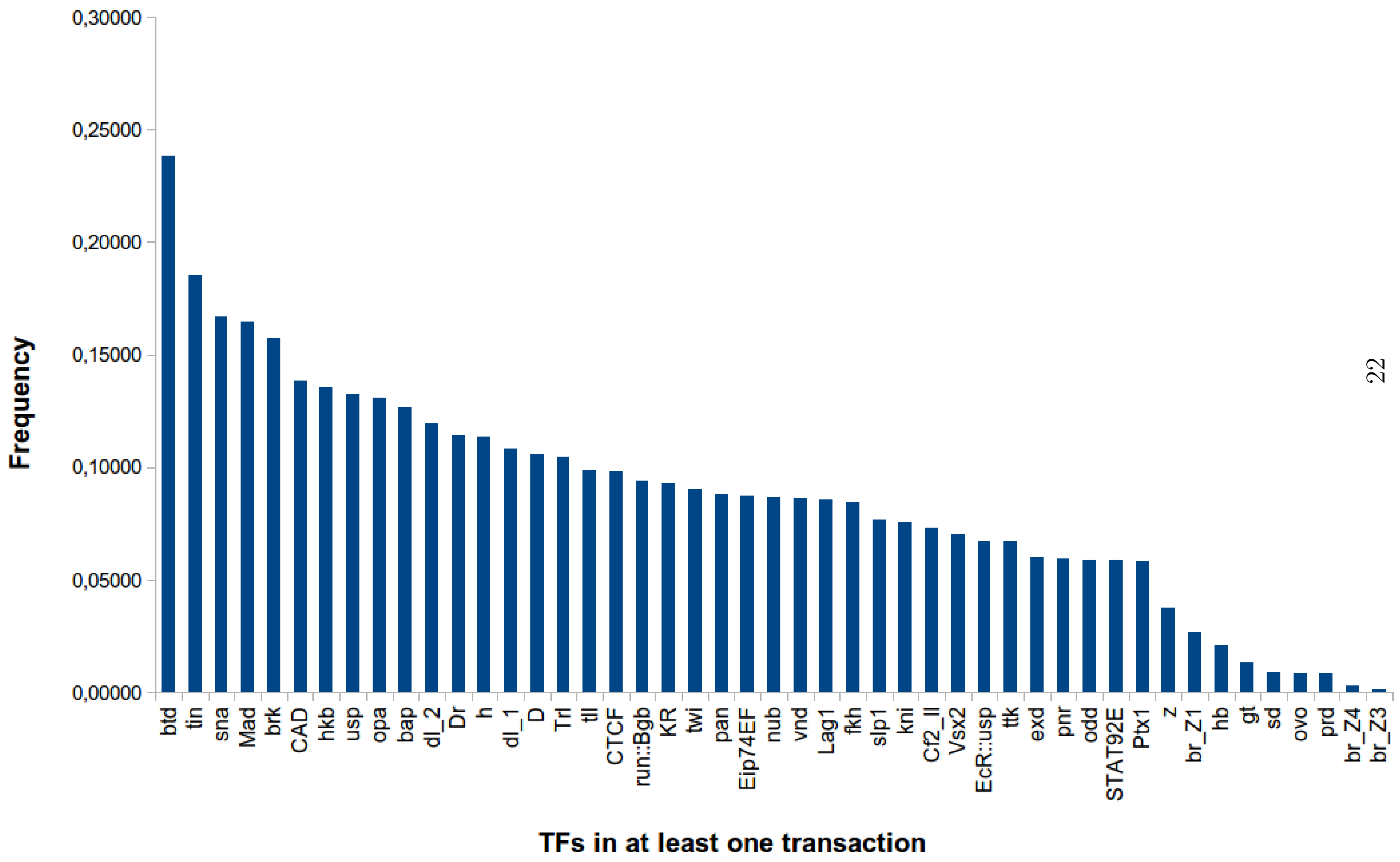

4.4 Complete result set. Third dataset.

Complete set of the combinations obtained when using the TFBSs detected by Patser. Each combination appears linked to its corresponding PubMed search (Drosophila genome), along with the related STRING graph, if available.

Id	Transcription factors	p-value	Support	String
1	*btd, hkb*	1.11×10^{-16}	0.045	
2	*btd, Mad*	1.11×10^{-16}	0.041	
3	*btd, opa*	1.11×10^{-16}	0.033	
4	*btd, h*	1.11×10^{-16}	0.003	
5	*Mad, brk*	1.11×10^{-16}	0.029	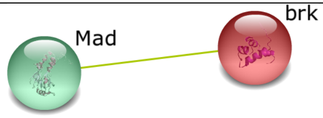
6	*btd, CTCF*	1.11×10^{-16}	0.023	
7	*Mad, opa*	1.11×10^{-16}	0.023	
8	*Mad, hkb*	1.11×10^{-16}	0.022	
9	*brk, opa*	1.11×10^{-16}	0.021	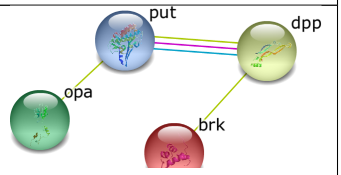
10	*Mad, h*	1.11×10^{-16}	0.019	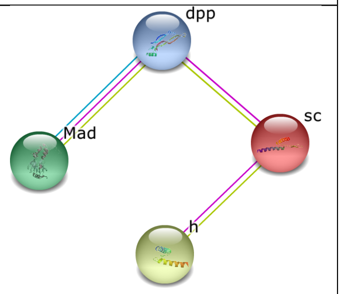
11	*hkb, opa*	1.11×10^{-16}	0.019	
12	*brk, h*	1.11×10^{-16}	0.018	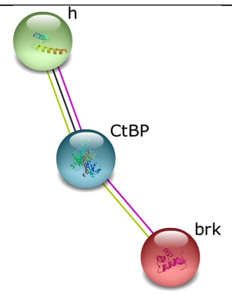
13	*btd, usp*	1.11×10^{-16}	0.017	
14	*Mad, Trl*	1.11×10^{-16}	0.017	

15	hkb, h	1.11×10^{-16}	0.017	
16	$Mad, CTCF$	1.11×10^{-16}	0.016	
17	$brk, CTCF$	1.11×10^{-16}	0.015	
18	opa, h	1.11×10^{-16}	0.015	
19	tin, vnd	1.11×10^{-16}	0.013	
20	$hkb, CTCF$	1.11×10^{-16}	0.013	
21	$opa, CTCF$	1.11×10^{-16}	0.013	
22	$h, CTCF$	1.11×10^{-16}	0.011	
23	dl_2, dl_1	1.11×10^{-16}	0.011	
24	btd, brk	1.91×10^{-13}	0.034	
25	brk, Trl	5.60×10^{-13}	0.015	
26	btd, KR	1.25×10^{-07}	0.019	
27	Mad, usp	4.35×10^{-07}	0.001	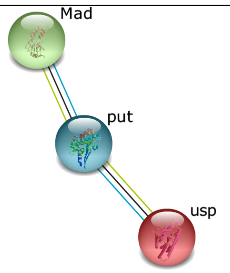
28	brk, hkb	1.23×10^{-06}	0.019	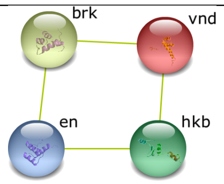
29	brk, pan	1.15×10^{-05}	0.011	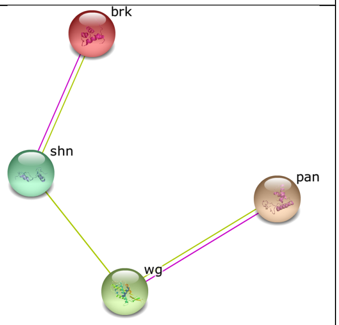
30	$btd, Lag1$	1.07×10^{-04}	0.017	

31	sna, h	1.86×10^{-04}	0.016	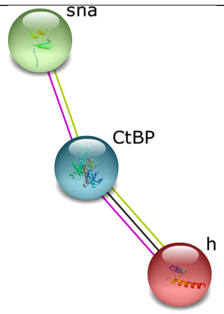
32	$sna, CTCF$	1.94×10^{-04}	0.013	
33	Mad, twi	2.24×10^{-04}	0.013	
34	opa, dl_1	4.06×10^{-04}	0.012	
35	btd, dl_1	8.39×10^{-04}	0.022	
36	btd, Trl	8.68×10^{-04}	0.021	
37	tin, h	1.60×10^{-03}	0.017	
38	opa, dl_2	3.85×10^{-03}	0.013	
39	btd, twi	6.54×10^{-03}	0.018	
